# The role of effectors in nonhost resistance to filamentous plant pathogens

**DOI:** 10.3389/fpls.2014.00582

**Published:** 2014-11-05

**Authors:** Remco Stam, Sophie Mantelin, Hazel McLellan, Gaëtan Thilliez

**Affiliations:** ^1^Division of Plant Sciences, University of Dundee – The James Hutton InstituteDundee, UK; ^2^Cell and Molecular Sciences, The James Hutton InstituteDundee, UK

**Keywords:** effectors, nonhost resistance, filamentous plant pathogens, oomycetes, fungi

## Abstract

In nature, most plants are resistant to a wide range of phytopathogens. However, mechanisms contributing to this so-called nonhost resistance (NHR) are poorly understood. Besides constitutive defenses, plants have developed two layers of inducible defense systems. Plant innate immunity relies on recognition of conserved pathogen-associated molecular patterns (PAMPs). In compatible interactions, pathogenicity effector molecules secreted by the invader can suppress host defense responses and facilitate the infection process. Additionally, plants have evolved pathogen-specific resistance mechanisms based on recognition of these effectors, which causes secondary defense responses. The current effector-driven hypothesis is that NHR in plants that are distantly related to the host plant is triggered by PAMP recognition that cannot be efficiently suppressed by the pathogen, whereas in more closely related species, nonhost recognition of effectors would play a crucial role. In this review we give an overview of current knowledge of the role of effector molecules in host and NHR and place these findings in the context of the model. We focus on examples from filamentous pathogens (fungi and oomycetes), discuss their implications for the field of plant-pathogen interactions and relevance in plant breeding strategies for development of durable resistance in crops.

## INTRODUCTION

In nature, successful pathogens are the exception, as the majority of plants are resistant to most pests and pathogen species. This form of disease resistance is known as nonhost resistance (NHR) and can be defined as resistance exhibited by an entire plant species to all genetic variants of a non-adapted pathogen species or forma specialis (f. sp.). Multiple factors contribute to NHR to unadapted pathogens, including constitutive defenses and induced defense mechanisms that result in plant immunity (Uma et al., 2011; Fan and Doerner, 2012).

Plant structure and chemistry form the first barriers encountered by any filamentous plant pathogen. The pathogen must locate a potential host. Variation in chemical compounds released by the plants can affect the attraction process (Morris and Ward, 1992). Once in contact, spores must germinate and form appressoria in order to penetrate the tissues. Both these processes are partly mediated by plant chemical components in compatible interactions (Ruan et al., 1995). Finally, the pathogen needs to find an appropriate source of nutrients in its host and thus the metabolic status of the plant can selectively determine its host/nonhost status (Stuttmann et al., 2011).

If the pathogen is able to overcome these barriers, it will face induced plant defenses. Most pathogens are defeated following detection of conserved pathogen molecules (Pathogen-Associated Molecular Patterns, PAMPs) by host cell surface pattern recognition receptors (PRRs) which activate pattern-triggered immunity (PTI; Zipfel, 2009). PAMPs are frequently parts of structural molecules that are essential for the pathogens and that cannot be readily changed to avoid their detection. However, adapted pathogens either evade recognition or suppress triggered plant defenses as summarized by the so-called Zigzag model (Jones and Dangl, 2006): PTI renders plants resistant, but pathogens deliver effector molecules thought to act in the apoplast or inside the plant cell, suppressing PTI (e.g., de Jonge et al., 2011; Wawra et al., 2012). In order to counter this, plants have a second layer of immune receptors encoded by resistance (*R*) genes, mainly nucleotide-binding – leucine-rich repeat (NB-LRR) proteins which upon activation, lead to effector-triggered immunity (ETI; Elmore et al., 2011). The ETI response is accompanied in most cases by a hypersensitive reaction (HR) – a localized programmed cell death – which is believed to prevent the spread of biotrophic pathogens from the infection site.

The role of plant receptors involved in both PTI and ETI as well as the pathogen effector arsenal are generally considered as the core components of the battleground in plant-pathogen interactions and NHR has been proposed to be largely based either on PTI in the absence of defense suppression, or on ETI from stacks of *R* genes (Schweizer, 2007; Niks and Marcel, 2009). Following this, a model of NHR was proposed by Schulze-Lefert and Panstruga (2011) that focused on inducible plant defenses and disregarded preformed defenses and physical cues. With respect to this condition, the model suggests that NHR in plants that are evolutionary distantly related to the natural host is predominantly triggered by PRR-mediated recognition, as PTI cannot be suppressed by the pathogen. By contrast, in more closely related species nonhost recognition of effectors and ETI are proposed to play a predominant role. In this review, we not only give an overview of current knowledge of the central role played by pathogen recognition systems and effectors in host and NHR but we also place these findings in the context of the NHR model. We focus on plant interaction with filamentous pathogens, and the relevance of NHR in plant breeding strategies for development of sustainable broad-spectrum resistance in crops.

## GENOMICS ADVANCES REVEAL EFFECTOR AND *R*-GENE DIVERSITY

Many genomes from filamentous phytopathogens have been sequenced, including both biotrophic and necrotrophic oomycetes (Haas et al., 2009; Baxter et al., 2010; Levesque et al., 2010) and numerous fungi with different lifestyles (Dean et al., 2005; Ma et al., 2010; Spanu et al., 2010; de Wit et al., 2012). Genome-scale analysis reveals that large numbers of putative effector genes are present in these phytopathogens. Some effectors occur in large families and the best-characterized examples are the RXLR and crinkling and necrosis (CRN) effectors in the oomycete genus *Phytophthora*. These families comprise 100s of genes (Haas et al., 2009; Shen et al., 2013; Stam et al., 2013). In fungi such large effector families with a common, defining, sequence motif appear to be absent. Nonetheless 100s of individual effectors and smaller effector families have been identified, including the conserved LysM effectors that protect *Cladosporium* spp. against chitin-associated defense responses (Bolton et al., 2008; Kombrink and Thomma, 2013), divergent families of cell wall degrading enzymes (CWDE; Ma et al., 2010; Spanu et al., 2010), clusters of putative cytoplasmic effectors (Saunders et al., 2012) and diverse families of Candidates for Secreted Effector Proteins (CSEPs) in the barley powdery mildew fungus (Pedersen et al., 2012).

The different mechanisms through which genomic and effector diversity within and between species can occur have recently been reviewed (e.g., Gladieux et al., 2014; Stukenbrock and Croll, 2014). Genome analyses show that many phytopathogens have a distinct genomic make-up. Nearly all show specific clustering patterns of genes. Isochore-like regions, which are CG-rich and non-coding, have been identified in the Ascomycota fungus *Leptosphaeria maculans* (Rouxel et al., 2011). The few genes present in these regions show important variation between populations. *Verticillium dahliae* genomes show characteristics of chromosomal reshuffling and harbor lineage-specific regions (LS) flanking chromosomal breakpoints. These LS are enriched for retrotransposons and other repetitive sequence elements (de Jonge et al., 2013). In the oomycete *P. infestans* a similar phenomenon has been described, where gene-dense regions are interspersed with gene-poor regions (Raffaele et al., 2010b). Effectors are frequently located in these “plastic” genomic regions. This observation prompted the hypothesis that this configuration allows for rapid effector diversification, thus allowing the pathogen to adapt to rapidly changing environments and to overcome resistance, a process also referred to as the two-speed-genome (Haas et al., 2009; Raffaele et al., 2010a; Raffaele and Kamoun, 2012; Karasov et al., 2014).

Similarly, as more plant genomes are sequenced, it is possible to compare their *R*-gene composition. These analyses show that the number of predicted *R* genes varies considerably from one species to another, even taking relative genome size into account: e.g., 54 in papaya (∼370 MB; Porter et al., 2009); 149 in *Arabidopsis* (∼125 MB; Arabidopsis Genome Initiative, 2000; Meyers, 2003); *ca.* 500 in rice (∼400 MB; Monosi et al., 2004; Rice Genome Project, 2005). Reported numbers are likely to be an under-estimate of the *R* genes present in each genome; the use of an enrichment technology (RenSeq) allows targeted sequencing, focusing on the NB-LRR composition. Using RenSeq, the number of predicted *R* genes present in the potato genome increased from 438 predicted in the original genome sequence, to 755 after enrichment (Jupe et al., 2012, 2013).

These genome studies suggest that effectors and *R* genes are under evolutionary pressure. Indeed, signatures of positive selection have been shown for effectors (Win et al., 2007), their targets (Kaschani et al., 2010), and *R* genes in *Arabidopsis* spp. (Mondragon-Palomino et al., 2002; Chen et al., 2010). However, comparative genomic studies as described above do not directly prove that effectors and *R* genes play roles in pathogen host range or nonhost recognition.

## EFFECTOR RECOGNITION IN HOST AND NONHOST PLANTS

The oomycete *Phytophthora infestans* causes disease in potato and tomato but it is unable to colonize the related solanaceous crop plant pepper (*Capsicum annum*). A screen of 54 *P. infestans* RXLR proteins in pepper revealed that many effectors are recognized in various pepper lines leading to a HR (7 on average and up to 36 in some accessions; Lee et al., 2014). Given that *P. infestans* has a predicted RXLR effector complement of >500 sequences and that 65% of the effectors tested in this study are detected and trigger a HR, this strongly suggests that NHR to *P. infestans* may be determined by recognition of multiple effector proteins. Similarly, a screen of 34 RXLR proteins from *Bremia lactucae* in 152 breeding lines of lettuce (*Lactuca sativa*) showed recognition of multiple effectors (Stassen et al., 2013). Two of these, BLG01 and BLG003, were recognized in 52 and two lines respectively. In addition, recent work on *P. capsici* has correlated NHR in a range of *Nicotiana* species with HR elicited by a single effector, PcAvr3a-like (Vega-Arreguín et al., 2014). Moreover, *Magnaporthe oryzae* formae speciales are only pathogenic on their original host species and as such are reproductively isolated. Thus, artificial crosses between these pathogens allow the analysis of the hybrid progeny to identify genes restricting host range. Among these genes, some were identified that encode secreted effector proteins belonging to the PWL family, variants of which are expected to be recognized in nonhost plants (Kang et al., 1995; Sweigard et al., 1995; Tosa et al., 2006).

Besides the direct evidence that multiple effectors can be recognized in nonhost plants, genetic studies of resistance in cereals and lettuce to different filamentous pathogens highlighted that NHR is based on multiple quantitative trait loci (QTLs; Jafary et al., 2008; Jeuken et al., 2008; Zhang et al., 2009; Aghnoum and Niks, 2010). In barley, QTLs associated with NHR showed similarity in location with QTLs for basal resistance to *Puccinia hordei* (Jafary et al., 2008). Interestingly, these QTLs show different and overlapping specificities and contain several putative *R* genes, suggesting that effector recognition might still play a role in this resistance. Besides, Zellerhoff et al. (2010) found that the differentially expressed genes in barley in response to closely related pathogens do not hugely differ between host and nonhost interactions but show distinct responses between the various pathogens. Interestingly, they also found a small cluster of genes overlapping between species, with similar functional annotations as those responsive to basal resistance. Unfortunately, the limited number of genes studied and the absence of pathogen data in this study, make it impossible to draw firm conclusions about the role of effector recognition in these interactions.

One way for a pathogen to avoid ETI is to lose recognized effectors. Whereas most effectors are thought to serve important functions during infection and seem to be indispensable, loss of effectors does not always lead to reduced fitness, most likely due to functional redundancy. For example, *P. striiformis* lacking effectors recognized by the wheat resistance protein Yr2 perform equally well on susceptible and Yr2 plants (Sørensen et al., 2013). Presence/absence polymorphisms are also common in *Phytophthora* RXLR and CRN effectors and in some cases, effector loss does not obviously affect fitness (Shan et al., 2004). A precursor to complete loss of effectors might be transgenerational gene silencing of effectors, as observed in *P. sojae* (Qutob et al., 2013). Thus, loss of recognized effectors in pathogen populations that are exposed to plants with cognate *R* genes shows that effector recognition plays a crucial role in avoiding resistance.

## HOST SPECIFIC EFFECTOR FUNCTION

In order for an effector to achieve its function and promote virulence, it is expected to manipulate one or more host target proteins or processes. One of the main ways effectors are thought to act is by suppressing defense responses activated during PTI. While there is a lot of evidence in the literature for effectors of filamentous pathogens suppressing immunity in host plants (de Jonge et al., 2011; Stassen and Van den Ackerveken, 2011) very little is known about effector function in distantly related nonhost plants, although CWDE from different phytopathogenic fungi show strongest enzymatic activity on their respective hosts (King et al., 2011). Recently, a protoplast expression system was used to identify RXLR effectors from *P. infestans* that were able to suppress PTI responses in a host (tomato) and in distantly related nonhost plant (*Arabidopsis*; Zheng et al., 2014). Of the 33 RXLR effectors screened, eight are able to suppress *FRK1* (*FLAG22-INDUCED RECEPTOR-LIKE KINASE 1*) induction triggered by the PAMP flg22 in tomato. Interestingly, only three of the eight maintained this activity in *Arabidopsis*, suggesting the failure of the remaining five effectors to successfully manipulate their targets in the nonhost plant. Unfortunately, the effector targets in this case are yet not known.

Antonovics et al. (2013) described how failure of infection of a nonhost plant by pathogens could be an incidental by-product of ongoing antagonistic evolution between adapted host and pathogens, a process they call non-evolved resistance. In agreement with this theory, recent work by Dong et al. (2014) demonstrated how effectors from two phylogenetically closely related oomycetes, *P. infestans* and *P. mirabilis*, evolved to specifically inhibit proteases from the distantly related plants tomato and four o’clock flower (*Mirabilis jalapa*) respectively. Cysteine proteases are involved in immunity and cell death signaling in plants (Gilroy et al., 2007; Shindo and van der Hoorn, 2007) and are known targets of effectors from fungi (Shabab et al., 2008; Mueller et al., 2013), oomycetes (Tian et al., 2006) and nematodes (Lozano-Torres et al., 2012). The effector *Pi*EPIC1 from *P. infestans* is a cysteine protease inhibitor, which interacts with the proteases PIP1 and RCR3 in tomato. The homologue in *P. mirabilis* (*Pm*EPIC1) is under diversifying selection compared to *Pi*EPIC1. The genes corresponding to the equivalent proteases of tomato RCR3 were cloned from potato and four o’clock flower (*Mirabilis RCR3-like protease 2, MRP2*) and the inhibitory activity of each effector was analyzed. *Pi*EPIC1 was able to efficiently suppress potato and tomato RCR3 activity but not MRP2 and the opposite was observed for *Pm*EPIC1 (Dong et al., 2014). This supports the hypothesis that pathogens may be unable to suppress immunity in distantly related nonhost plants through failure of effectors to correctly manipulate their plant targets.

## UNDERSTANDING EFFECTOR ACTIVITY FOR NONHOST RESISTANCE BREEDING IN CROPS

Studies presented in this review support the fact that effector recognition plays an important role in resistance or in susceptibility between closely related plant species. In this light, *R* genes from related resistant crop species have been used for many years in breeding programs to provide resistance to specific isolates of a given pathogen species. Resistance genes from distantly related species have also been successfully introduced. For example, the introduction of the barley *R* gene *MLA1* in immunocompromised *Arabidopsis* mutants provided resistance to *Blumeria graminis* f. sp. *hordei* (Maekawa et al., 2012). Similarly, introduction of the tomato *Ve1* gene in *Arabidopsis* conferred resistance to *Verticillium* spp. (Fradin et al., 2011). This shows that the mechanism involved downstream of the recognition event can be conserved between plant species, and even between monocotyledonous and dicotyledonous plants. Consequently the transfer of *R* genes from a distantly related nonhost into a host plant could be used as a new source of resistance (Wulff et al., 2011). However, deployment of a single *R* gene in a variety usually leads to resistance breakdown in the field within a couple of years and merely serves as an example of rapid adaptations that phytopathogens are capable of in natural and agricultural systems (Fry, 2008; Palloix et al., 2009; Stukenbrock and McDonald, 2009).

Transcriptomics studies of barley powdery mildew on immunocompromised *Arabidopsis* mutants revealed large effector sets that were similarly expressed early during compatible and incompatible interactions, but a reduction of effector expression in the presence of an active *MLA1 R* gene was observed (Hacquard et al., 2013). This suggests that a large number of effectors might be essential during infection and a subset of them may be recognized in nonhost interactions. The identification of indispensable effectors and their cognate *R* genes could be a straightforward step toward resistance breeding in crops. However, recent studies show that a single point mutation in either NB-LRR or effector genes can alter the *R*-gene specificity or the effector function (Brunner et al., 2010; Segretin et al., 2014; Stirnweis et al., 2014).

Alternative breeding strategies include modification of host genes targeted by effectors that are essential for the pathogen to establish itself, in order to artificially create the incompatibility observed in divergent host species. These genes are sometimes referred to as susceptibility (*S*) genes (Vogel et al., 2002; Pavan et al., 2010; Lapin and Van den Ackerveken, 2013). Modification of *S* genes is thought to be more durable; however, changing host proteins to avoid effector binding but retain normal functionality might prove to be very difficult (van Schie and Takken, 2014).

## CONCLUSION

NHR is by definition more durable than host resistance; we have summarized here the recent findings supporting the idea that NHR is likely to be governed by multiple recognition events. However, effector recognition in distantly related nonhost plants has not frequently been observed. Taken together, the recent studies presented here rather support the original model of NHR proposed by Schulze-Lefert and Panstruga (2011). To determine involvement of effectors in NHR in distantly related crops, more comprehensive population genomics and transcriptomics studies will be required to advance our understanding of effector occurrence and expression within and between hosts and nonhosts as well as in pathogen populations. More studies are also required to examine effector function and recognition in both closely and distantly related nonhost plants. This work might help answer whether PTI does indeed play a greater role in NHR in more distal plant species. Alternatively it may find that the inability of the pathogen to modify crucial host processes due to lack of coevolution, in other words, the non-evolved resistance might already happen in closely related hosts. Additionally, we have highlighted two approaches that can be used to breed resistant plants. Whereas both approaches are very different, in both cases the study of effector proteins will be instrumental toward understanding the infection process and selecting appropriate target genes.

## Conflict of Interest Statement

The authors declare that the research was conducted in the absence of any commercial or financial relationships that could be construed as a potential conflict of interest.
